# Are Mindfulness and Self-Compassion Related to Psychological Distress and Communication in Couples Facing Lung Cancer? A Dyadic Approach

**DOI:** 10.1007/s12671-016-0602-0

**Published:** 2016-09-06

**Authors:** Melanie P. J. Schellekens, Johan C. Karremans, Miep A. van der Drift, Johan Molema, Desiree G. M. van den Hurk, Judith B. Prins, Anne E. M. Speckens

**Affiliations:** 1 Radboud Centre for Mindfulness, Department of Psychiatry, Radboud University Medical Centre, Reinier Postlaan 4, P.O. Box 9101, 6500HB Nijmegen, The Netherlands; 2 Behavioural Science Institute, Radboud University, Nijmegen, The Netherlands; 3 Department of Lung Diseases, Radboud University Medical Centre, Nijmegen, The Netherlands; 4 Department of Medical Psychology, Radboud University Medical Centre, Nijmegen, The Netherlands

**Keywords:** Mindfulness, Self-compassion, Lung cancer, Psychological distress, Partner communication, Actor-partner interdependence model

## Abstract

Lung cancer patients and their spouses report high rates of distress. Due to the increasing popularity of and evidence for mindfulness-based interventions in cancer, mindfulness and self-compassion have been identified as potentially helpful skills when coping with cancer. This dyadic study examined how mindfulness and self-compassion are related to psychological distress and communication about cancer in couples facing lung cancer. Using the actor-partner interdependence model, self-reported mindfulness, self-compassion, psychological distress and communication about cancer were analyzed in a cross-sectional sample of 88 couples facing lung cancer. Regarding psychological distress, no difference was found between patients and spouses. In both partners, own levels of mindfulness (*B* = −0.19, *p* = .002) and self-compassion (*B* = −0.45, *p* < .001) were negatively related to own distress levels. At a dyadic level, own self-compassion was less strongly associated with distress if the partner reported high self-compassion (*B* = 0.03, *p* = .049). Regarding communication about cancer, patients reported to communicate more openly with their partner than with spouses. However, after controlling for gender, this difference was no longer significant. In both partners, own self-compassion (*B* = 0.03, *p* = .010) was significantly associated with own communication while mindfulness was not. A trend showed that mindfulness of the partner was related to more open communication in the individual (*B* = 0.01, *p* = .080). These findings give a first indication that mindfulness and self-compassion skills may go beyond the individual and could impact couple functioning. Future research should examine whether couples facing (lung) cancer may benefit from programs in which mindfulness and self-compassion are cultivated.

## Introduction

Receiving a diagnosis of lung cancer has a major impact. Patients with lung cancer develop severe physical symptoms, undergo radical treatment and face a poor prognosis. As lung cancer is strongly associated with smoking, patients often feel stigmatized and tend to blame themselves and are blamed by others for developing cancer (Chapple et al. 2004; Else-Quest et al. 2009; Milbury et al. 2012), which has a negative impact on their wellbeing. In fact, lung cancer patients are more likely to meet the threshold of psychological distress (23–63 %) than are patients with other types of cancer (Carlson et al. 2004; Gao et al. 2010; Linden et al. 2012). Not only patients but also their spouses can be heavily affected by the lung cancer diagnosis. Factors contributing to heightened distress include dealing with practical tasks, such as coordinating the patient’s medical care, managing the patient’s emotional reactions to the illness and coping with an uncertain future (Mosher et al. 2013). In fact, the rates of distress among spouses tend to be similar to those of lung cancer patients (Mosher et al. 2013; Ostlund et al. 2010), although in general, females report the highest rates of distress irrespective of being patient or spouse (Hagedoorn et al. 2008).

Most studies have examined the factors associated with psychological and relational distress for patients and their spouses separately. Yet, partners in long-term relationships are interdependent, mutually affecting each other (Kelley & Thibaut 1978). In couples facing lung cancer, the coping of one partner presumably affects the extent to which the other partner is able to cope. A meta-analysis of 35 studies on couples coping with various types of cancer (*n*_*couples*_ = 2468) revealed that psychological distress levels between cancer patients and spouses are moderately associated with one another (*r* = .29), supporting the notion that couples coping with cancer respond as an interdependent emotional system rather than as two separate individuals (Hagedoorn et al. 2008). Such results emphasize the importance of taking a dyadic approach when studying the functioning of couples facing lung cancer. In dyadic studies, individual as well as partner factors are examined simultaneously in both patients and spouses, while taking their interdependency into account (Kenny et al. 2006). In the current study, we refer to a patient’s partner as the spouse and use the term partner to refer to members of the couple in general.

There are some findings from previous dyadic studies with couples coping with lung cancer. For instance, a longitudinal study with 158 couples showed that behavioural disengagement (i.e. giving up the attempt to cope), blaming the patient for having cancer, caregiver-related health problems and relationship maintenance behaviour (e.g. engaging in shared tasks) affected one’s own, and often also the other partner’s, psychological distress and/or dyadic adjustment (Badr and Carmack Taylor 2008; Carmack Taylor et al. 2008; Milbury et al. 2012, 2013). Thus, in line with the notion of interdependence, such findings suggest that if one partner has difficulty in coping with the cancer diagnosis, it can negatively affect the other partner’s coping ability as well.

So far, the exploration of what factors may possibly protect patients and spouses from developing distress remains limited. Two potentially protective factors that may help couples cope with a lung cancer diagnosis are mindfulness skills and self-compassion. Mindfulness is defined as intentionally paying attention in a non-judgmental way to present moment experiences (Kabat-Zinn 1990). In essence, mindfulness can best be considered a state of awareness (Bishop et al. 2004), although there are individual differences in the extent to which one is generally mindful to present moment experiences (i.e. as a trait or skill). Self-compassion involves acknowledging one’s pain and recognizing this is part of the human experience while meeting the pain with kindness and understanding (Neff 2003). It has been argued that mindfulness is strongly related to self-compassion as paying mindful attention to painful experiences promotes the ability to actively comfort oneself and remember that painful experiences are part of being human (Neff and Dahm 2014). However, mindfulness and self-compassion do not always co-arise. One can non-judgmentally accept present moment thoughts, emotions and sensations, without actively soothing oneself or having a sense of common humanity. In other words, mindfulness is aimed at the experience itself, whereas self-compassion is aimed at the experiencer and includes feelings of kindness and common humanity not entailed by mindfulness alone (Germer 2009; Neff and Dahm 2014). Both these factors are receiving increasing scientific attention, partly due to the popularity and effectiveness of mindfulness-based interventions in both healthy as chronically ill populations, including cancer patients (Piet et al. 2012). A rapidly increasing and large body of research has now demonstrated that mindfulness and self-compassion can have powerful effects on individual functioning and well-being, particularly in stressful situations (for an overview, see Creswell and Lindsay 2014).

Two important outcomes that might be affected by the protective ability of mindfulness skills and self-compassion are psychological distress and communication about cancer. When people experience psychological distress, they can respond quite automatically, by ruminating about the past and worrying about the future (Carlson and Speca 2010). Cultivating the ability to mindfully turn towards one’s (distressing) thoughts and emotions while having a non-judgemental and accepting attitude can help acknowledge and allow these thoughts and emotions for what they are without getting immersed in them. Not directly reacting to thoughts and emotions but attending to them with open and accepting awareness can facilitate coping with stress and promotes taking better care of oneself (Segal et al. 2002). Supporting this notion, there is good evidence that, in general, self-reported mindfulness skills are related to less psychological distress, e.g. Baer et al. (2008). Also, there is some initial evidence that, in cancer patients, increases in mindfulness skills are related to decreases in stress symptoms (Birnie et al. 2010). Likewise, being compassionate towards the suffering that a cancer diagnosis may cause can reduce the additional distress that often results from self-blame and self-judgment (Neff 2003). In general, self-reported self-compassion has been significantly related to psychological well-being outcomes, such as less depression and anxiety and greater life satisfaction (Neff 2003). Also, among cancer patients, self-compassion has been related to less psychological distress (Przezdziecki et al. 2013).

A recent review showed that in couples facing cancer, the quality of communication between partners has important implications for their psychological and relational wellbeing (Traa et al. 2015). While open communication helps buffering relationship distress, protecting the other partner by avoiding communication of one’s own worries and fears about the cancer can increase both partners’ distress (Manne et al. 2007). Studies on communication about cancer have also shown gender differences overruling role differences, with female patients and female partners perceiving poorer communication within the family than do their male counterparts (Lim et al. 2014). A prerequisite for effective communication is that partners are able to recognize and identify their emotions and thoughts. Whereas denial and suppression of emotions often is a natural response when facing difficulties, mindful awareness of current experiences should allow an individual to be better ‘in touch’ with his or her internal psychological state. Similarly, approaching difficult feelings with self-compassion may facilitate to identify and face these emotions more easily. By becoming aware of and acknowledging one’s own and one’s partner’s pain, without judging oneself or the other for it, partners may be more likely to consciously choose to discuss one’s regrets, hopes and fears (Segal et al. 2002). In this manner, mindfulness and self-compassion may facilitate more effective communication with the partner.

There are some initial findings supporting this reasoning. Wachs and Cordova (2007) showed that self-reported mindfulness was associated with increased ability to identify and communicate emotions to the partner, which in turn promoted marital satisfaction. Similarly, Barnes et al. (2007) found that self-reported mindfulness was positively correlated with more constructive communication patterns between partners during a conflict discussion in the lab. Also, some studies suggest that self-compassion promotes communication. Yarnell and Neff (2013) found that self-reported self-compassion was positively associated with the tendency to communicate about (rather than to subordinate) one’s own needs during conflict with the partner. Finally, a dyadic study by Neff and Beretvas (2013) demonstrated that partners with relatively high levels of self-reported self-compassion were described by their spouses as being more caring, emotionally connected, accepting and autonomy-supporting while being less detached, controlling and importantly, less verbally aggressive than those lacking self-compassion.

In sum, there is some initial evidence that mindfulness and self-compassion are associated with more effective coping with distress and improved communication about distress. However, most studies that examined the role of mindfulness and self-compassion in partner relationships focused on the individual (e.g. Burpee and Langer 2005; Wachs and Cordova 2007; Yarnell and Neff 2013; for an overview, see Kozlowski 2013). Although informative, such studies have not addressed how the mindfulness skills and self-compassion of one partner may be related to the psychological and relational functioning of the other partner. As noted, coping with distress, and coping with lung cancer in particular, is something that concerns not only the patient but both partners’ coping abilities, potentially affecting each other mutually. The few dyadic studies that have examined mindfulness and self-compassion between partners show mixed findings regarding whether the mindfulness skills or self-compassion of one partner affect the functioning of the other partner (Barnes et al. 2007; Neff and Beretvas 2013; Pakenham and Samios 2013; Williams and Cano 2014). As recently proposed based on a review of the literature (Karremans et al. 2015), dyadic studies are needed to understand whether and how mindfulness and self-compassion play a role in the functioning of partner relationships.

In addition, research on the role of mindfulness skills and self-compassion within partner relationships mostly has focused on non-distressed couples (e.g. Burpee and Langer 2005; Carson et al. 2004; Neff and Beretvas 2013; Wachs and Cordova 2007), with the exception of a study on couples facing multiple sclerosis (Pakenham and Samios 2013) and a study with chronic pain patients (Williams and Cano 2014). The latter study found that spousal mindfulness skills were associated with higher perceived partner support in the patient. However, whether mindfulness and self-compassion promote dyadic coping in cancer patients and their spouses has not been studied yet. It can be reasoned that being able to respond mindfully and compassionately to both one’s own and the partner’s distress may affect a couple particularly during highly distressing episodes in life, such as when one partner has been diagnosed with lung cancer.

The aim of the present study was (1) to examine whether mindfulness skills and self-compassion are associated with lower psychological distress and better communication about cancer in patients with lung cancer and their spouses and (2) to explore whether the mindfulness skills and self-compassion of one partner might be associated with the psychological distress and communication about cancer of the other partner. To address these research questions, we examined whether, in both patients and spouses, higher levels of own mindfulness skills and own self-compassion would be associated with lower levels of own psychological distress and with higher levels of own communication about cancer. We then explored whether the mindfulness skills and self-compassion of one partner would be associated with lower psychological distress and better communication about cancer in the other partner. Finally, we explored whether each partner’s mindfulness skills and self-compassion would moderate those of the other partner for each outcome variable. For example, one partner in a couple with high levels of mindfulness and self-compassion may compensate for the other partner’s lack of mindfulness and self-compassion, possibly buffering distress and promoting communication in both partners (cf. Vohs et al. 2011). Given previously found gender differences in distress and communication (Hagedoorn et al. 2008; Lim et al. 2014), we controlled for possible gender differences.

## Method

### Participants

The majority of participants were selected from a consecutive sample of lung cancer patients and partners that participated in a systematic screening study for psychiatric disorders (Schellekens et al. 2016) (between March 2013 and March 2014). This sample was supplemented with the (pre-randomisation) baseline scores of lung cancer patients and spouses that participated in a randomized controlled trial (RCT) on the effectiveness of mindfulness-based stress reduction (MBSR) in patients with lung cancer and their partners (Schellekens et al. 2014) (between March 2012 and March 2013). Both the screening study and RCT have been approved by our ethical review board CMO Arnhem-Nijmegen and are registered under number 2011-519.

Included were patients who (a) were diagnosed with cytologically or histologically proven non-small cell lung cancer or small cell lung cancer and (b) completed or were still receiving treatment. Excluded were patients who (a) were younger than 18 years of age or (b) were not able to understand or use the Dutch language. For the current study, we selected only data of patients and spouses when both patient and spouse filled in the questionnaires.

With an expected population correlation of around 0.3 for the outcome variable, psychological distress, between patients and spouses (based on our pilot study, van den Hurk et al. 2015), data from 80 dyads were needed to have 80 % power to detect a medium-sized difference with an alpha of 0.05 (Kenny et al. 2006).

### Procedure

In both studies, a nurse practitioner called patients and partners at least 1 month after diagnosis to explain the study procedure. Patients and/or spouses who were willing to participate were contacted separately by a researcher, who sent them an information leaflet and a consent form. After informed consent was received, the participants filled out the questionnaires.

### Measures

#### Demographic, relationship and clinical characteristics

Demographic (gender, age, educational level) and relationship characteristics (marital status, sexual orientation, relationship length) were assessed. Relationship satisfaction was measured with the ten-item Satisfaction subscale of the Investment Model Scale (IMS-S) (Rusbult et al. 1998) on which participants can score how satisfied they are with different aspects of their relationship. Total scores can range between 1 (totally not satisfied) and 8 (totally satisfied). Chart reviews were conducted to determine disease characteristics (stage of disease, date of diagnosis at the time of study enrolment and current anti-cancer treatment).

#### Mindfulness Skills

The Dutch-validated 24-item short form of the Five Facet Mindfulness Questionnaire (FFMQ-SF; Bohlmeijer et al. 2011) is a reliable and valid alternative to the original FFMQ (Baer et al. 2008), which is based on an exploratory factor analysis of five mindfulness measures to provide an empirical integration of these independent attempts to operationalize mindfulness. The FFMQ-SF can be divided into five subscales: observing, describing, acting with awareness, non-judging of inner experience and non-reactivity to inner experience. As applied in other studies (e.g. Bowlin and Baer 2012; Josefsson et al. 2011), we used the total scale of the FFMQ-SF in the analyses. A sample item is “I perceive my feelings and emotions without having to react to them”. Internal consistency for the total scale was 0.74 in the present study.

#### Self-Compassion

The Dutch-validated 12-item short form of the Self-Compassion Scale (SCS-SF; Raes et al. 2011) is a reliable and valid alternative to the original SCS (Neff 2003). Items include “I try to be understanding and patient towards those aspects of my personality I don’t like” and “I’m disapproving and judgmental about my own flaws and inadequacies”. In the present study, internal consistency of the total scale was 0.74.

#### Psychological Distress

The Hospital Anxiety and Depression Scale (HADS) (Spinhoven et al. 1997; Zigmond and Snaith 1983) was developed to measure psychological distress in somatic patient populations and consists of an anxiety and depression subscale. A sample item is “I feel tense or wound up”. The HADS has been validated in several Dutch patient populations and oncology patients and showed good internal consistency (Bjelland et al. 2002; Spinhoven et al. 1997). Internal consistency of the total scale was 0.89 in the present study.

#### Communication About Cancer

We (MS, AS) translated the 18-item Mutual Interpersonal Sensitivity scale (MIS) into Dutch with a forward backward translation (Lewis et al. 2008). It measures the extent to which one communicates about the cancer with the partner. It consists of two nine-item subscales: open communication and avoiding negative thoughts about the cancer. Items include “We are comfortable sharing feelings about the lung cancer with each other”. In the present study, the internal consistency of the total scale was 0.88.

### Data Analyses

Patients included in the dyadic dataset were compared with those not included, using independent sample *t* tests and *χ* ^2^ tests. Moreover, included patients and partners that were recruited via the screening study were compared with patients and partners recruited via the RCT. To characterize the final sample, the means and standard deviations of the participants’ characteristics and major study variables were calculated for patients and partners separately. Dependent sample *t* tests were conducted to examine differences between patient and partner scores on the major study variables. Pearson correlation of the major study variables within patients and partners were calculated. Partial correlations examined the interdependence between patients and partners.

**Fig. 1:**
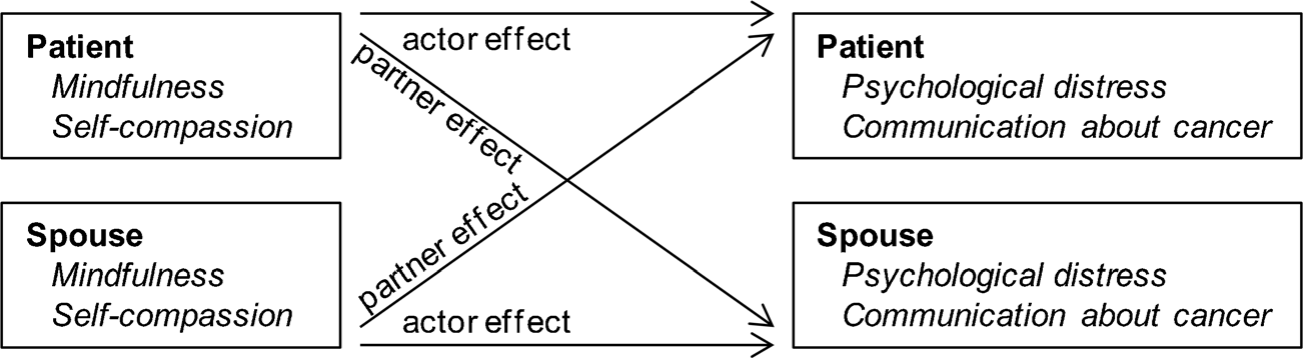
Actor-partner interdependence model of actor and partner mindfulness and actor and partner self-compassion in psychological distress and communication about cancer. Note that actor and partner effects are independent of whether the individual is a patient or a spouse

Multilevel modelling in SPSS version 20 on a pairwise dataset was performed to examine the role of actor and partner effects of mindfulness and self-compassion on psychological distress and communication about cancer in couples coping with lung cancer, by using the actor-partner interdependence model (APIM) (Kenny et al. 2006). Although not all couples are married, we refer to the patient’s partner as the spouse and use the term partner to refer to members of the couple in general. The APIM is designed to analyze dyadic processes (Kenny et al. 2006) and has been used to examine illness adjustment in several dyads, including lung cancer patients and their spouses (e.g. Badr and Carmack Taylor 2008). As data from partners within a couple are related, the analyses must model the interdependence between partners *and* adjust for this interdependence to prevent bias in the statistical tests. By means of a multilevel modelling approach, the APIM takes into account the interdependence by treating data of the two partners as nested within the couple. In the APIM, each person’s outcome (irrespective of whether the person is a patient or a spouse) is associated with (1) his or her own score on the predictor variable, referred to as *actor effects* (e.g. partner A’s level of mindfulness associated with partner A’s level of distress), and (2) his or her partner’s score on the predictor variable, referred to as *partner effects* (e.g. partner B’s level of mindfulness associated with partner A’s level of distress). See Fig. 1 for a depiction of the APIM.

The linear mixed model included the couple as unit of analysis at the upper level and partners within the couple at the lower level to estimate each outcome variable separately (psychological distress, communication about cancer) as a function of whether the subject is a patient or a spouse (coded as 1 and −1 respectively; referred to as cancer role), the predictors of interest of the actor and partner (mindfulness, self-compassion) and possible interactions. First, we ran a mindfulness model (including mindfulness but no self-compassion predictors) and a self-compassion model (including self-compassion but no mindfulness predictors) for each outcome. We re-ran the models after trimming non-significant interaction terms to provide more stable estimates for the main effects, showing similar findings as the full model for each predictor regarding direction and significance. Next, the actor and partner effects of mindfulness and self-compassion and contributing interactions with *p* values <.10 were carried forward to a combined model. In addition, the models were controlled for covariates (gender, age, relationship length, relationship satisfaction, cancer stage, time since diagnosis), showing that only gender substantively changed the interpretation of the model communication about cancer, which was therefore added to both models (males coded as 1 and females coded as −1). To indicate to what extent continuous predictors are correlated with outcome, the effect sizes for each significant continuous predictor were calculated. In accord with the APIM model, these are partial correlations, using the formula $r=\sqrt{t^2/\left({t}^2+df\right)}$ (Kenny et al. 2006).

Since the correlation between mindfulness and self-compassion within participants was large (*r*(169) = .59, *p* < .001), we performed ordinary least squares regression with actor mindfulness and actor self-compassion as predictors to obtain the indices of the impact of multicollinearity on the precision of estimation. For each outcome variables, we found a variance inflation factor below the cut-off of 10 (i.e. 1.6) and a tolerance value higher than the cut-off of 0.1 (i.e. 0.6), indicating that multicollinearity did not threaten the stability of the estimates (Cohen et al. 2003).

## Results

Of the 165 lung cancer patients who were eligible for the present study, 88 patients and 88 partners were included. Patients were excluded because they did not have a spouse (*n* = 35), their spouse did not participate (*n* = 34), patients did not fill out the questionnaires (*n* = 6) or for an unknown reason (*n* = 2). The 88 patients did not differ from the patients who were excluded from the present study on demographic, relationship and clinical characteristics and study variables. Patients and partners participating in the screening study (*n*_*couples*_ *=* 67) did not differ from patients and partners participating in the RCT (*n*_*couples*_ = 21) on demographic characteristics and study variables. However, patients from the screening study did appear to be more often in the curative stage of the disease (64 versus 38 %) and participated sooner after diagnosis (*M* = 2.3 months [SD = 13.1] versus *M* = 11.6 months [SD = 1.6]) than did patients from the RCT. Patient and spouse characteristics can be found in Table 1. Patients and partners were together for 34.9 years (SD = 14.3) and were generally satisfied with their relationship (*M =* 6.4; SD = 1.5). Based on their treatment, the majority of patients were in the curative stage of the disease (58 %) and the mean time since diagnosis was 4.5 months (SD = 7.6). Patients in the curative disease stage did not differ from palliative patients on any of the study variables. Descriptive statistics of mindfulness, self-compassion, psychological distress and communication about cancer for patients and spouses can be found in Table 2. Except for communication about the cancer, patients and spouses did not differ from each other. Interestingly, patients reported a better communication about the cancer with their partner than spouses (*t*(78) = 2.80, *p* = .006). Based on the cut-off levels of our screening study (HADS-T ≥ 15; Schellekens et al. 2016), 26 patients (29.5 %) and 31 spouses (31.8 %) reported clinically heightened levels of psychological distress.

**Table 1:** Demographic, relationship and clinical characteristics of patients (*n* = 88) and spouses (*n* = 88)

	Patients	Spouses
	*n* (%)	*n* (%)
Gender		
Male	59 (67)	28 (32)
Female	29 (33)	60 (68)
Age, *M* (SD)	62.8 (8.2)	61.6 (8.4)
Educational level^a^		
Low	34 (39)	27 (31)
Intermediate	30 (34)	33 (38)
High	22 (25)	23 (26)
Marital status		
Married	82 (93)	82 (93)
Living together	6 (7)	6 (7)
Sexual orientation		
Opposite sex	87 (99)	87 (99)
Same sex	1 (1)	1 (1)
Relationship length, *M* (SD)	34.9 (14.3)	34.9 (14.3)
Relationship satisfaction (IMS-S), *M* (SD)	6.5 (1.5)	6.2 (1.5)
Stage of disease (curative/palliative)	51/37 (58/42)	
I	25 (28)	
II	15 (17)	
IIIa	16 (18)	
IIIb	11 (13)	
IV	21 (24)	
Months since diagnosis, *M* (SD)	4.5 (7.6)	
Current treatment	34 (19)	
Chemotherapy	24 (14)	
Radiotherapy	6 (3)	
Chemotherapy and radiotherapy	4 (2)	

*IMS-S* satisfaction subscale of investment model scale

^a^Low educational level = primary and lower secondary education; intermediate = upper secondary education; high = higher vocational training and university.

**Table 2:** Mean and standard deviation of study variables in patients (*n* = 88) and spouses (*n* = 88)

Variables	Potential range	Patients	Spouses	*p* ^a^
		*M* (SD)	*M* (SD)	
Mindfulness skills (FFMQ-SF)	24–120	81.3 (10.4)	81.4 (9.6)	.839
Self-compassion (SCS-SF)	6–42	28.1 (5.6)	27.9 (4.4)	.854
Psychological distress (HADS)	0–42	11.8 (7.4)	12.4 (6.8)	.550
Communication about cancer (MIS)	1–5	3.9 (0.7)	3.7 (0.7)	.006

*FFMQ-SF* short form of Five Facet Mindfulness Questionnaire, *SCS-SF* short form of Self-Compassion Scale, *HADS* Hospital Anxiety and Depression Scale, *MIS* Mutual Interpersonal Sensitivity scale

^a^Dependent sample *t* test

### Associations Within and Between Patient and Spouse Scores

In the patients (Table 3), both mindfulness and self-compassion are significantly related to a lower level of psychological distress (*r*(83) = −.49, *p* < .001; *r*(82) = −.55, *p* < .001, respectively) and a better communication about the cancer (*r*(80) = .33, *p* = .003; *r*(80) = .34, *p* = .002, respectively). In spouses (Table 3), both mindfulness and self-compassion were related to lower levels of psychological distress (*r*(85) = −.43, *p* < .001; *r*(85) = −.42, *p* < .001, respectively) but not to communication about cancer (*r*s < .18, ns). In sum, both higher mindfulness and self-compassion were related to lower distress in both patients and spouses and related to better quality of communication about the cancer, but only in patients.

**Table 3:** Correlations of the study variables within and between patients (*n* = 88) and spouses (*n* = 88)

		Variables	1	2	3	4
A. Correlations of the study variables within patients (*n* = 88)						
1		Mindfulness skills (FFMQ-SF)	**–**			
2		Self-compassion (SCS-SF)	.62**	**–**		
3		Psychological distress (HADS)	−.49**	−.55**	**–**	
4		Communication about cancer (MIS)	.33**	.34**	−.10	**–**
B. Correlations of the study variables within spouses (*n* = 88)						
1		Mindfulness skills (FFMQ-SF)	**–**			
2		Self-compassion (SCS-SF)	.55**	**–**		
3		Psychological distress (HADS)	−.43**	−.42**	**–**	
4		Communication about cancer (MIS)	.10	.18	−.29*	**–**
C. Correlations of the study variables between patients (*n* = 88) and spouses (*n* = 88)						
		Variables	Patients			
1		Mindfulness skills (FFMQ-SF)	.26*	.06	−.09	.10**
2	Spouses	Self-compassion (SCS-SF)	.21†	.22*	−.21*	.09
3		Psychological distress (HADS)	−.22*	−.15	.25*	−.12
4		Communication about cancer (MIS)	.23*	.10	−.02	.43**

*FFMQ-SF* short form of Five Facet Mindfulness Questionnaire, *SCS-SF* short form of Self-Compassion Scale, *HADS* Hospital Anxiety and Depression Scale, *MIS* Mutual Interpersonal Sensitivity scale

*p < .05; **p < .01; †*p* < .10

Paired-samples correlations (see Table 3) showed that the scores were significantly associated for mindfulness (*r*(82) = .26, *p* = .016), self-compassion (*r*(81) = .22, *p* = .041), psychological distress (*r*(86) = .25, *p* = .020) and communication about cancer (*r*(77) = .43, *p* < .001), implying their interdependence. Controlling for gender, partial Pearson correlations showed that patient and spouse scores remained significantly correlated for all variables.

### Actor-Partner Interdependence Model: Psychological Distress

Table 4 shows the results of the mindfulness and self-compassion models for psychological distress. No significant difference between patients’ and spouses’ psychological distress was found in none of the three models. Post hoc controlling for gender did not change the interpretation of the effects in any of the models. Only in the self-compassion model, gender was a marginally significant predictor, indicating that males tended to report less psychological distress than females do.

**Table 4:** Multi-level models estimating actor and partner effects of (1) mindfulness and (2) self-compassion on psychological distress and communication about cancer, controlling for gender in couples coping with lung cancer (*n* = 88)

	Psychological distress					Communication about cancer				
	B	SE	95 % CI	*t*	Effect size: *r*	B	SE	95 % CI	*t*	Effect size: *r*
Mindfulness model										
Intercept	12.32	0.53	11.26 to 13.38	23.12		3.80	0.06	3.67 to 3.93	58.99	
Gender	−0.77	0.50	−1.75 to 0.22	−1.54		0.06	0.05	−0.03 to 0.16	1.34	
Cancer role	−0.04	0.47	−0.99 to 0.90	−0.09		0.08	0.05	−0.02 to 0.17	1.66	
Actor mindfulness	−0.32	0.05	−0.42 to −0.22	−6.29**	0.45	0.01	0.01	0.00 to 0.02	1.91†	0.15
Partner mindfulness	−0.04	0.05	−0.14 to 0.06	−0.74		0.01	0.01	0.00 to 0.02	1.75†	0.14
Self-compassion model										
Intercept	12.05	0.51	11.03 to 13.07	23.48		3.80	0.06	3.67 to 3.92	59.66	
Gender	−0.91	0.48	−1.87 to 0.04	−1.91†		0.04	0.05	−0.05 to 0.14	0.93	
Cancer role	0.07	0.47	−0.86 to 1.00	0.15		0.08	0.05	−0.01 to 0.17	1.85†	
Actor self-compassion	−0.68	0.10	−0.87 to −0.49	−7.01**	0.49	0.03	0.01	0.01 to 0.06	3.23**	0.25
Partner self-compassion	−0.14	0.10	−0.34 to 0.05	−1.49		0.01	0.01	−0.01 to 0.02	0.74	
Actor self−compassion × partner self-compassion	0.03	0.01	0.00 to 0.06	1.94†	0.21					

Effect size $r=\sqrt{t^2/\left({t}^2+df\right)}$. Gender is coded 1 (males) and −1 (females). Cancer role is coded 1 (patients) and −1 (spouses).

*B* unstandardised coefficient, *SE* standard error, *CI* confidence interval

*p < .05; **p < .01; †*p* < .10

**Fig. 2:**
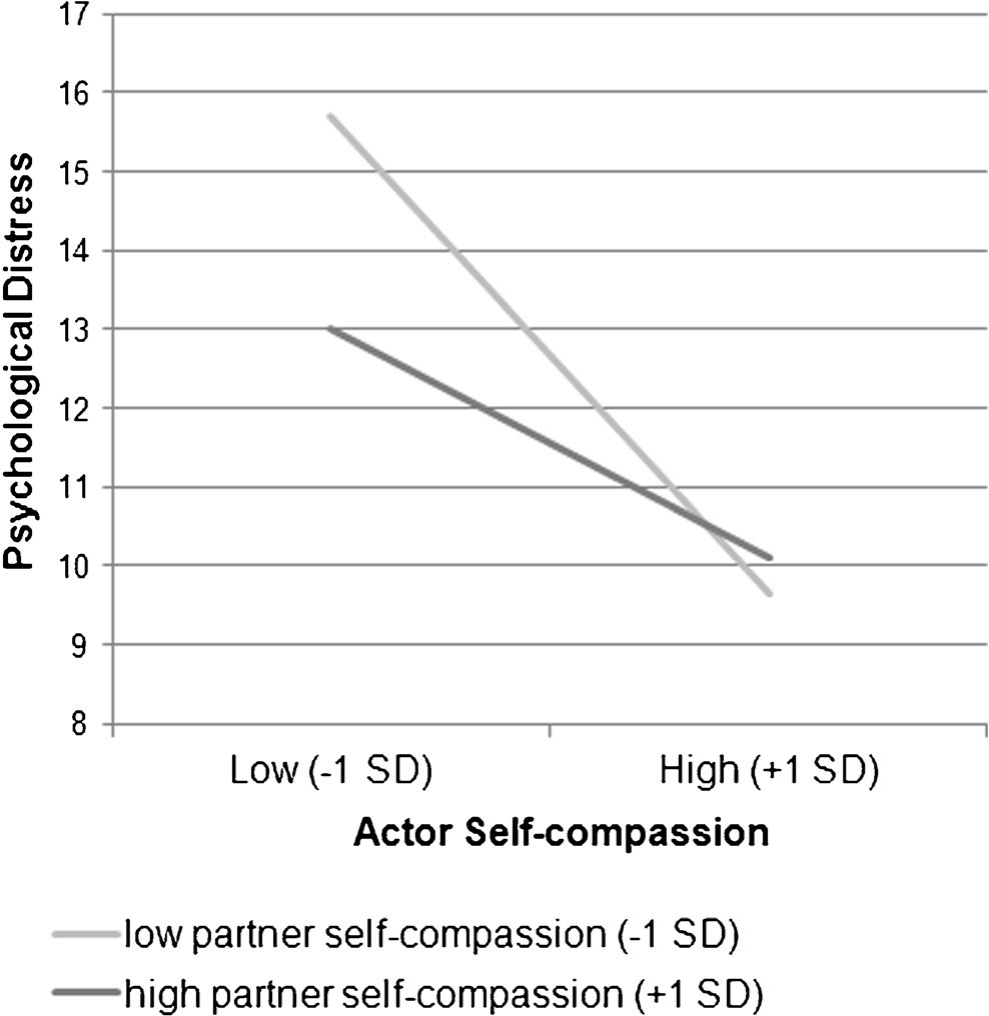
The partner effect of self-compassion moderated the relationship between actor self-compassion and actor psychological distress. High actor and partner effects of self-compassion correspond with 1 SD above the mean and low actor and partner effects of self-compassion correspond with 1 SD below the mean

In the mindfulness model, as the previous correlational analyses already indicated, we did find a significant actor effect of mindfulness (*B* = −0.32, *t*(156) = −6.29, *p* < .001), indicating that higher scores on own mindfulness were related to lower scores on own psychological distress. No partner effect of mindfulness was found. In the self-compassion model, we found a significant actor effect of self-compassion (*B* = −0.68, *t*(158) = −7.01, *p* < .001), indicating that higher scores on own self-compassion were related to lower scores on own psychological distress. However, no partner effect of self-compassion was found. Yet, a marginally significant interaction effect was found between actor and partner self-compassion scores (*B* = 0.03, *t*(80) = 1.94, *p* = .056). Specifically, when the self-compassion of the partner is relatively high, the association between own self-compassion and psychological distress tended to be weaker. In the combined model (Table 5), the actor effects of mindfulness (*B* = −0.19, *t*(151) = −3.13, *p* = .002) and self-compassion (*B* = −0.45, *t*(155) = −3.86, *p* < .001) remained significant, demonstrating that higher own mindfulness and self-compassion scores were related to lower own psychological distress scores. In addition, the interaction between actor and partner self-compassion scores became significant (*B* = 0.03, *t*(78) = 2.00, *p* = .049). As displayed in Fig. 2, the association between own self-compassion and psychological distress was weaker when the self-compassion of the partner was relatively high.

**Table 5:** Multi-level model estimating actor and partner effects of mindfulness and self-compassion on psychological distress and communication about cancer, controlling for gender in couples coping with lung cancer (*n* = 88)

Combined model	Psychological distress					Communication about cancer				
	B	SE	95 % CI	*t*	Effect size: *r*	B	SE	95 % CI	*t*	Effect size: *r*
Intercept	12.12	0.50	11.12 to 13.11	24.28		3.80	0.06	3.67 to 3.93	59.19	
Gender	−0.73	0.49	−1.69 to 0.24	−1.49		0.06	0.05	−0.04 to 0.15	1.17	
Cancer role	−0.04	0.47	−0.96 to 0.89	−0.08		0.08	0.05	−0.01 to 0.17	1.78^†^	
Actor mindfulness	−0.19	0.06	−0.31 to −0.07	−3.13^**^	0.25	<0.01	0.01	−0.01 to 0.01	<0.01	
Partner mindfulness	−0.01	0.06	−0.13 to 0.11	−0.15		0.01	0.01	0.00 to 0.02	1.76^†^	0.14
Actor self-compassion	−0.45	0.12	−0.68 to −0.22	−3.86^**^	0.30	0.03	0.01	0.01 to 0.06	2.60^*^	0.21
Partner self-compassion	−0.11	0.12	−0.34 to 0.12	−0.96		−0.01	0.01	−0.03 to 0.02	−0.40	
Actor self-compassion × partner self-compassion	0.03	0.02	<0.01 to 0.06	2.00^*^	0.22					

Effect size $r=\sqrt{t^2/\left({t}^2+df\right)}$. Gender is coded 1 (males) and −1 (females). Cancer role is coded 1 (patients) and −1 (spouses)

*B* unstandardised coefficient, *SE* standard error, *CI* confidence interval

*p < .05; **p < .01; †*p* < .10

### Actor-Partner Interdependence Model: Communication About Cancer

Table 4 shows the results of the mindfulness and self-compassion models for communication about cancer. Cancer role was a significant predictor of communication about cancer in all three models, such that patients reported to communicate significantly more with their partner than spouses did. However, after controlling for gender, this effect was no longer significant in any of the models, suggesting that the difference between patients and spouses might be explained by more spouses being females.

In the mindfulness model, there were marginally significant actor (*B* = 0.01, *t*(152) = 1.91, *p* = .058) and partner effects (*B* = 0.01, *t*(152) = 1.75, *p* = .082) of mindfulness, suggesting that higher scores on mindfulness of oneself and of the partner tended to be associated with higher scores of own communication about cancer. In the self-compassion model, actor self-compassion (*B* = 0.03, *t*(151) = 3.23, *p* = .002) is a significant predictor of communication about cancer, demonstrating that higher scores on own self-compassion were related to higher scores on own communication about the cancer. We did not find a significant partner effect of self-compassion on communication. In the combined model (Table 5), the actor effect of mindfulness was no longer marginally significant. Yet, the partner effect of mindfulness remained marginally significant (*B* = 0.01, *t*(150) = 1.76, *p* = .080), indicating that the higher mindfulness scores of the partner tended to be associated with higher scores of own communication about the cancer. The actor effect of self-compassion remained significant (*B* = 0.03, *t*(147) = 2.60, *p* = .010), suggesting that higher own self-compassion scores were related to higher own communication about cancer scores.

## Discussion

The goal of the present research was to explore the roles of mindfulness and self-compassion in couples facing lung cancer. Several findings should be highlighted. First, a person’s levels of mindfulness and self-compassion were negatively related to his or her psychological distress level. These findings are consistent with studies showing that self-reported mindfulness skills and self-compassion are related to less psychological distress in cancer populations (Garland et al. 2013; Przezdziecki et al. 2013) as well as in other populations (Baer et al. 2008; Neff 2003; Pakenham and Samios 2013).

Second, and extending these findings, we also found some indication of partner effects: the association between self-compassion and psychological distress in each individual in the couple depends on the partner’s level of self-compassion. Specifically, the higher the partner’s level of self-compassion, the weaker the association between own level of self-compassion and psychological distress. This may suggest that when one partner displays less self-compassion, the other partner may compensate by showing more compassion, which alleviates the distress in both partners. As self-compassion may not always come as naturally to people (Feldman and Kuyken 2011), taking turns in showing more and less self-compassion may be an efficient way for couples to cope with a severe illness. Or, put differently, having at least one partner with high levels of self-compassion in the relationship may compensate for a lack of self-compassion in the other partner. These results are in line with previous research findings, showing that complementarity of coping styles (e.g. acceptance of illness, protective buffering) facilitated couples’ adjustment (Badr 2004; Pakenham and Samios 2013).

Third, regarding communication about the cancer, a number of effects were obtained. We found a difference between patients and partners regarding communication about cancer, but controlling for gender, this effect became non-significant. This suggests that the difference between patients and spouses might be partially explained by more spouses being female, such that a female spouse might perceive less quality and frequency of communication than their male counterparts. Previous studies have shown that gender differences can overrule role differences, with female patients and female spouses perceiving poorer communication within the family than males do (Lim et al. 2014). Future research could further explore this difference between males and females and whether there is an interaction with cancer role (i.e. patients versus spouses). Additionally, a persons’ level of self-compassion was related to better communication about the cancer. This finding is in line with previous literature showing that self-compassion is associated with more constructive communication and relationship satisfaction (Neff and Beretvas 2013; Yarnell and Neff 2013). Interestingly, we found a trend that own levels of mindfulness tended to be associated with a better communication about cancer but when actor and partner self-compassion were added to the model, this trend disappeared. This might suggest that self-compassion is possibly more important for communicating about cancer than mindfulness skills are. Moreover, and importantly, we found some evidence for a partner effect (albeit marginally), such that individuals tended to communicate more openly about the cancer when the *partner* was relatively high (versus low) in mindfulness. Possibly, when partners anticipate that the other is better able to “handle” communication about distressing thoughts or feelings, they communicate more openly about distress related to the cancer. This is in contrast with previous findings on healthy couples by Barnes et al. (2007), who found that mindfulness was related to better communication only within the individual, not between partners.

As a recent meta-analysis concluded, to better understand how coping with cancer operates within couples, dyadic studies taking the interdependency between partners into account have the greatest validity and clinical utility in this field (Regan et al. 2015). By using multilevel modelling on a pairwise dataset, we could not only examine associations within individuals but also between partners. Both self-compassion and mindfulness of the partners affected the individual in ways that would remain obscured when examining their respective roles in distress and communication only at the level of the individual. The present findings underline the importance of taking a *dyadic* approach when examining coping mechanisms in couples with cancer. Such findings were obtained with an adequate number of couples based on a predefined power analysis. As noted, dyadic studies on coping in couples facing cancer or other diseases are relatively scarce.

Despite these strengths of the study, a few limitations should be noted. The cross-sectional design prevents us from drawing conclusion about the causal relationship between the predictors, mindfulness and self-compassion, and the outcome variables, psychological distress and communication about cancer. Future studies should adopt a dyadic approach in longitudinal designs to enable researchers to draw conclusions about whether mindfulness and self-compassion actually benefit couples facing lung cancer in the long run. In addition, since the majority of patients in the current sample were in the curative stage of the disease, the study sample is less representative of the global lung cancer population as reported by the global cancer statistics (Jemal et al. 2011). This might be indicated by the large number of patients who are referred to the Radboud University Medical Centre in the early stage of the disease to undergo surgery. Moreover, several couples did not participate in the study because their spouses declined participation, mainly because they were physically impaired or they felt participation would be too stressful. Due to the omission of these possibly more distressed couples, the range of variables might be truncated, implicating that true relationships might be even stronger than presently reported. Another limitation is that we solely relied on self-report questionnaires for assessing mindfulness skills and self-compassion. While the validity of these measures is under debate (Grossman and Van Dam 2011; Muris and Petrocchi 2016), the scales have high internal consistency and have been adopted successfully in studies on the effects of mindfulness (Baer 2011). Although the FFMQ and SCS measure closely related concepts, which could threaten the stability of the estimates, the statistical test showed no sign of multicollinearity. In addition, due to shared method variance (Orth 2013), the chances of finding actor effects are higher than finding partner effects because the actor effect is based on data from a common source (e.g. mindfulness level from actor predicts distress level from actor) while the partner effect is based on data from different sources (e.g. mindfulness level from partner predicts distress level from actor). Future research examining mindfulness skills and self-compassion in the APIM should therefore consider using both self- as well as partner-reported questionnaires.

In sum, this is one of the first dyadic studies on mindfulness and self-compassion in couples coping with cancer. We found some preliminary evidence that more self-compassion in the partner was related to less distress in individuals with low levels of self-compassion, and the results suggest that having a mindful partner tends to promote the other partner’s willingness to communicate about the cancer. These findings point to the possibility that mindfulness and self-compassion skills go beyond the individual and may impact couple functioning. In addition, our findings suggest that couples facing (lung) cancer may benefit from programs aimed at improving mindfulness skills and self-compassion. In fact, MBSR and Mindfulness-Based Cognitive Therapy (MBCT) (Segal et al. 2002) have proven to be effective in reducing anxiety and depressive symptoms in cancer patients (Piet et al. 2012). Although self-compassion also seems to increase after MBSR/MBCT (Birnie et al. 2010; Kuyken et al. 2010), its focus lies on the cultivation of mindfulness rather than self-compassion (Kabat-Zinn 1990; Labelle et al. 2014). However, as the present findings suggest that self-compassion might play an at least as important role as mindfulness skills in the dyadic coping of couples facing lung cancer, an intervention focused on the cultivation of (self-)compassion might also be a worthwhile possibility, e.g. compassion-focused therapy (Gilbert 2009), mindful self-compassion (Neff and Germer 2013), mindfulness-based compassionate living (van den Brink and Koster 2015). Future trials could examine the effectiveness of mindfulness-based and self-compassion-based interventions for couples facing cancer.

## References

[CR1] Badr H (2004). Coping in marital dyads: a contextual perspective on the role of gender and health. Personal Relationships.

[CR2] Badr H, Carmack Taylor CL (2008). Effects of relationship maintenance on psychological distress and dyadic adjustment among couples coping with lung cancer. Health Psychology.

[CR3] Baer RA (2011). Measuring mindfulness. Contemporary Buddhism.

[CR4] Baer RA, Smith GT, Lykins E, Button D, Krietemeyer J, Sauer S (2008). Construct validity of the Five Facet Mindfulness Questionnaire in meditating and nonmeditating samples. Assessment.

[CR5] Barnes S, Brown KW, Krusemark E, Campbell WK, Rogge RD (2007). The role of mindfulness in romantic relationship satisfaction and responses to relationship stress. Journal of Marital and Family Therapy.

[CR6] Birnie K, Speca M, Carlson LE (2010). Exploring self-compassion and empathy in the context of mindfulness-based stress reduction (MBSR). Stress and Health.

[CR7] Bishop SR, Lau M, Shapiro S, Carlson LE, Anderson ND, Carmody J (2004). Mindfulness: a proposed operational definition. Clinical Psychology: Science and Practice.

[CR8] Bjelland I, Dahl AA, Haug TT, Neckelmann D (2002). The validity of the hospital anxiety and depression scale: an updated literature review. Journal of Psychosomatic Research.

[CR9] Bohlmeijer E, ten Klooster PM, Fledderus M, Veehof M, Baer RA (2011). Psychometric properties of the Five Facet Mindfulness Questionnaire in depressed adults and development of a short form. Assessment.

[CR10] Bowlin SL, Baer RA (2012). Relationships between mindfulness, self-control, and psychological functioning. Personality and Individual Differences.

[CR11] Burpee LC, Langer EJ (2005). Mindfulness and marital satisfaction. Journal of Adult Development.

[CR12] Carlson LE, Speca M (2010). Mindfulness-based cancer recovery.

[CR13] Carlson LE, Angen M, Cullum J, Goodey E, Koopmans J, Lamont L (2004). High levels of untreated distress and fatigue in cancer patients. British Journal of Cancer.

[CR14] Carmack Taylor CL, Badr H, Lee JH, Fossella F, Pisters K, Gritz ER (2008). Lung cancer patients and their spouses: psychological and relationship functioning within 1 month of treatment initiation. Annals of Behavioral Medicine.

[CR15] Carson JW, Carson KM, Gil KM, Baucom DH (2004). Mindfulness-based relationship enhancement. Behavior Therapy.

[CR16] Chapple A, Ziebland S, McPherson A (2004). Stigma, shame, and blame experienced by patients with lung cancer: qualitative study. British Medical Journal.

[CR17] Cohen J, Cohen P, West SG, Aiken LS (2003). Applied multiple regression/correlation analysis for the behavioural sciences.

[CR18] Creswell JD, Lindsay EK (2014). How does mindfulness training affect health? A mindfulness stress buffering account. Current Directions in Psychological Science.

[CR19] Else-Quest NM, LoConte NK, Schiller JH, Hyde JS (2009). Perceived stigma, self-blame, and adjustment among lung, breast and prostate cancer patients. Psychology & Health.

[CR20] Feldman C, Kuyken W (2011). Compassion in the landscape of suffering. Contemporary Buddhism.

[CR21] Gao W, Bennett MI, Stark D, Murray S, Higginson IJ (2010). Psychological distress in cancer from survivorship to end of life care: prevalence, associated factors and clinical implications. European Journal of Cancer.

[CR22] Garland SN, Tamagawa R, Todd SC, Speca M, Carlson LE (2013). Increased mindfulness is related to improved stress and mood following participation in a mindfulness-based stress reduction program in individuals with cancer. Integrative Cancer Therapies.

[CR23] Germer CK (2009). The mindful path to self-compassion.

[CR24] Gilbert P (2009). Introducing compassion-focused therapy. Advances in Psychiatric Treatment.

[CR25] Grossman P, Van Dam NT (2011). Mindfulness, by any other name…: trials and tribulations of sati in western psychology and science. Contemporary Buddhism.

[CR26] Hagedoorn M, Sanderman R, Bolks HN, Tuinstra J, Coyne JC (2008). Distress in couples coping with cancer: a meta-analysis and critical review of role and gender effects. Psychological Bulletin.

[CR27] Jemal A, Bray F, Center MM, Ferlay J, Ward E, Forman D (2011). Global cancer statistics. CA: A Cancer Journal for Clinicians.

[CR28] Josefsson T, Larsman P, Broberg A, Lundh L-G (2011). Self-reported mindfulness mediates the relation between meditation experience and psychological well-being. Mindfulness.

[CR29] Kabat-Zinn J (1990). Full catastrophe living: Using the wisdom of your body and mind to face stress, pain and illness.

[CR30] Karremans JC, Schellekens MPJ, Kappen G (2015). Bridging the sciences of mindfulness and romantic relationships: a theoretical model and research agenda. Personality and Social Psychology Review.

[CR31] Kelley, H. H., & Thibaut, J. W. (1978). Interpersonal relations: A theory of interdependence. New York: Wiley.

[CR32] Kenny DA, Kashy DA, Cook WL (2006). Dyadic data analysis.

[CR33] Kozlowski A (2013). Mindful mating: exploring the connection between mindfulness and relationship satisfaction. Sexual and Relationship Therapy.

[CR34] Kuyken W, Watkins E, Holden E, White K, Taylor RS, Byford S (2010). How does mindfulness-based cognitive therapy work?. Behaviour Research and Therapy.

[CR35] Labelle LE, Campbell TS, Faris P, Carlson LE (2014). Mediators of mindfulness-based stress reduction (MBSR): assessing the timing and sequence of change in cancer patients. Journal of Clinical Psychology.

[CR36] Lewis FM, Fletcher KA, Cochrane BB, Fann JR (2008). Predictors of depressed mood in spouses of women with breast cancer. Journal of Clinical Oncology.

[CR37] Lim J-w, Paek M, Shon E-j (2014). Gender differences in couple communication during cancer survivorship. Psycho-Oncology.

[CR38] Linden W, Vodermaier A, MacKenzie R, Greig D (2012). Anxiety and depression after cancer diagnosis: prevalence rates by cancer type, gender, and age. Journal of Affective Disorders.

[CR39] Manne SL, Norton TR, Winkel G, Ostroff JS, Fox K, Grana G (2007). Protective buffering and psychological distress among couples coping with breast cancer: the moderating role of relationship satisfaction. Journal of Family Psychology.

[CR40] Milbury K, Badr H, Carmack CL (2012). The role of blame in the psychosocial adjustment of couples coping with lung cancer. Annals of Behavioral Medicine.

[CR41] Milbury K, Badr H, Fossella F, Pisters KM, Carmack CL (2013). Longitudinal associations between caregiver burden and patient and spouse distress in couples coping with lung cancer. Supportive Care in Cancer.

[CR42] Mosher CE, Bakas T, Champion VL (2013). Physical health, mental health, and life changes among family caregivers of patients with lung cancer. Oncology Nursing Forum.

[CR43] Muris, P., & Petrocchi, N. (2016). Protection or vulnerability? A meta‐analysis of the relations between the positive and negative components of self‐compassion and psychopathology. *Clinical Psychology & Psychotherapy*. 10.1002/cpp.2005 26891943

[CR44] Neff KD (2003). The development and validation of a scale to measure self-compassion. Self and Identity.

[CR45] Neff KD, Beretvas SN (2013). The role of self-compassion in romantic relationships. Self and Identity.

[CR46] Neff KD, Dahm KA, Robinson M, Meier B, Ostafin B (2014). Self-compassion: what it is, what it does, and how it relates to mindfulness. Mindfulness and self-regulation.

[CR47] Neff KD, Germer CK (2013). A pilot study and randomized controlled trial of the mindful self-compassion program. Journal of Clinical Psychology.

[CR48] Orth U (2013). How large are actor and partner effects of personality on relationship satisfaction? The importance of controlling for shared method variance. Personality and Social Psychology Bulletin.

[CR49] Ostlund U, Wennman-Larsen A, Persson C, Gustavsson P, Wengstrom Y (2010). Mental health in significant others of patients dying from lung cancer. Psycho-Oncology.

[CR50] Pakenham KI, Samios C (2013). Couples coping with multiple sclerosis: a dyadic perspective on the roles of mindfulness and acceptance. Journal of Behavioral Medicine.

[CR51] Piet J, Wurtzen H, Zachariae R (2012). The effect of mindfulness-based therapy on symptoms of anxiety and depression in adult cancer patients and survivors: a systematic review and meta-analysis. Journal of Consulting and Clinical Psychology.

[CR52] Przezdziecki A, Sherman KA, Baillie A, Taylor A, Foley E, Stalgis-Bilinski K (2013). My changed body: breast cancer, body image, distress and self-compassion. Psycho-Oncology.

[CR53] Raes F, Pommier E, Neff KD, Van Gucht D (2011). Construction and factorial validation of a short form of the self-compassion scale. Clinical Psychology and Psychotherapy.

[CR54] Regan TW, Lambert SD, Kelly B, Falconier M, Kissane D, Levesque JV (2015). Couples coping with cancer: exploration of theoretical frameworks from dyadic studies. Psycho-Oncology.

[CR55] Rusbult CE, Martz JM, Agnew CR (1998). The investment model scale: measuring commitment level, satisfaction level, quality of alternatives, and investment size. Personal Relationships.

[CR56] Schellekens, M. P. J., van den Hurk, D. G. M., Prins, J. B., Molema, J., Donders, A. R. T., Woertman, W. H. et al., (2014) Study protocol of a randomized controlled trial comparing Mindfulness-Based Stress Reduction with treatment as usual in reducing psychological distress in patients with lung cancer and their partners: the MILON study. *BMC Cancer, 14*(1), 3. 10.1186/1471-2407-14-3 PMC3893473 24386906

[CR57] Schellekens, M. P. J., van den Hurk, D. G. M., Prins, J. B., Molema, J., van der Drift, M. A., & Speckens, A. E. M. (2016). The suitability of the Hospital Anxiety and Depression Scale, Distress Thermometer and other instruments to screen for psychiatric disorders in both lung cancer patients and their partners. *Journal of Affective Disorders, 203*, 176–183, doi:10.1016/j.jad.2016.05.044. 10.1016/j.jad.2016.05.044 27295374

[CR58] Segal ZV, Williams JMG, Teasdale JD (2002). Mindfulness-based cognitive therapy for depression: a new approach to preventing relapse.

[CR59] Spinhoven P, Ormel J, Sloekers PPA, Kempen G, Speckens AEM, VanHemert AM (1997). A validation study of the hospital anxiety and depression scale (HADS) in different groups of Dutch subjects. Psychological Medicine.

[CR60] Traa MJ, De Vries J, Bodenmann G, Den Oudsten BL (2015). Dyadic coping and relationship functioning in couples coping with cancer: a systematic review. British Journal of Health Psychology.

[CR61] van den Brink E, Koster F (2015). Mindfulness-Based Compassionate Living: a new training program to deepen mindfulness with heartfulness.

[CR62] van den Hurk, D. G. M., Schellekens, M. P. J., Molema, J., Speckens, A. E. M., & van der Drift, M. A. (2015). Mindfulness-Based Stress Reduction for lung cancer patients and their partners: Results of a mixed methods pilot study. Palliative Medicine, 29(7), 652-660, doi:10.1177/0269216315572720. 10.1177/0269216315572720 PMC4457793 25701663

[CR63] Vohs KD, Finkenauer C, Baumeister RF (2011). The sum of friends’ and lovers’ self-control scores predicts relationship quality. Social Psychological and Personality Science.

[CR64] Wachs K, Cordova JV (2007). Mindful relating: exploring mindfulness and emotion repertoires in intimate relationships. Journal of Marital and Family Therapy.

[CR65] Williams AM, Cano A (2014). Spousal mindfulness and social support in couples with chronic pain. Clinical Journal of Pain.

[CR66] Yarnell LM, Neff KD (2013). Self-compassion, interpersonal conflict resolutions, and well-being. Self and Identity.

[CR67] Zigmond AS, Snaith RP (1983). The hospital anxiety and depression scale. Acta Psychiatrica Scandinavica.

